# Global mapping of RNA homodimers in living cells

**DOI:** 10.1101/gr.275900.121

**Published:** 2022-05

**Authors:** Marta M. Gabryelska, Andrew P. Badrock, Jian You Lau, Raymond T. O'Keefe, Yanick J. Crow, Grzegorz Kudla

**Affiliations:** 1 MRC Human Genetics Unit, University of Edinburgh, Edinburgh, EH4 2XU, United Kingdom;; 2 Flinders Health and Medical Research Institute, College of Medicine and Public Health, Flinders University, Bedford Park, South Australia 5042, Australia;; 3 Centre for Genomic and Experimental Medicine, Institute of Genetics and Cancer, University of Edinburgh, Edinburgh, EH4 2XU, United Kingdom;; 4 Division of Evolution and Genomic Sciences, Faculty of Biology, Medicine and Health, School of Biological Sciences, University of Manchester, Manchester, M13 9PL, United Kingdom

## Abstract

RNA homodimerization is important for various physiological processes, including the assembly of membraneless organelles, RNA subcellular localization, and packaging of viral genomes. However, understanding RNA dimerization has been hampered by the lack of systematic in vivo detection methods. Here, we show that CLASH, PARIS, and other RNA proximity ligation methods detect RNA homodimers transcriptome-wide as “overlapping” chimeric reads that contain more than one copy of the same sequence. Analyzing published proximity ligation data sets, we show that RNA:RNA homodimers mediated by direct base-pairing are rare across the human transcriptome, but highly enriched in specific transcripts, including *U8* snoRNA, *U2* snRNA, and a subset of tRNAs. Mutations in the homodimerization domain of *U8* snoRNA impede dimerization in vitro and disrupt zebrafish development in vivo, suggesting an evolutionarily conserved role of this domain. Analysis of virus-infected cells reveals homodimerization of SARS-CoV-2 and Zika genomes, mediated by specific palindromic sequences located within protein-coding regions of *N* gene in SARS-CoV-2 and *NS2A* gene in Zika. We speculate that regions of viral genomes involved in homodimerization may constitute effective targets for antiviral therapies.

The biological functions of RNA molecules depend on their ability to form intra- and intermolecular interactions, often mediated by Watson-Crick base-pairing. Intramolecular base-pairing determines the structure and function of RNA, including rRNA and tRNA; it regulates viral replication; and it influences the efficiency of mRNA translation into proteins. Intermolecular RNA–RNA base-pairing underlies codon–anticodon recognition, splicing, and regulation of gene expression by miRNA and siRNAs. Intramolecular and intermolecular interactions are interdependent, and according to the competing endogenous RNA (ceRNA) hypothesis (Salmena et al. 2011; Gardiner et al. 2015), intermolecular RNA interactions have the potential to rewire regulatory networks and expand the information encoded in a genome.

An intermolecular interaction between two identical molecules is known as homodimerization. Although homodimers are common in proteins (Bergendahl and Marsh 2017), relatively few homodimers of RNA molecules have been described in vivo (for review, see Bou-Nader and Zhang 2020). Perhaps the best studied are dimers of the HIV genome, which are initiated by an interaction between two copies of the palindromic sequence known as DIS (Berkhout and van Wamel 1996). This interaction leads to the formation of an extended double helix that joins together two copies of the genome, launching a series of events that leads to the packaging of the pair of genomes into one capsid (Paillart et al. 2004). Homodimerization events have been described in retroviruses, hepatitis C virus, SARS coronavirus, and in bacteriophages (Clever et al. 2002; Shetty et al. 2010; Ishimaru et al. 2013; Dubois et al. 2018).

RNA oligomerization also plays a role in the process of phase separation, which leads to the formation of membraneless RNA-containing organelles, such as P-bodies, stress granules, nucleoli, Cajal bodies, and others (Jain and Vale 2017; Khong et al. 2017; Nguyen et al. 2018; Van Treeck et al. 2018; Van Treeck and Parker 2018). There is growing evidence that such granules are formed via transient protein–RNA and RNA–RNA interactions. As an example, homo- and heterodimerization of mRNA induces the formation of distinct types of phase-separated droplets in a filamentous fungus (Langdon et al. 2018). Homodimerization also influences the localization of *oskar* and *bicoid* mRNAs in *Drosophila* embryos (Ferrandon et al. 1997; Wagner et al. 2001, 2004; Jambor et al. 2011; Masliah et al. 2013). Strong interaction between mRNA and pre-mRNA of *CUP1* gene leads to RNA miscompartmentalization and localization to cytoplasmic foci, possibly including P-bodies and stress granules (Qu et al. 2014).

An example of pathogenic homodimerization has been observed in a mutated variant of a human mitochondrial tRNA (Wittenhagen and Kelley 2002; Roy et al. 2005). Additionally, tRNA fragments (tRFs) were shown to form homodimers (Tosar et al. 2018) and tetramers (Lyons et al. 2017). CAG and other repeats underlying RNA expansion disorders form hairpin structures, with a stem composed of periodically occurring standard C-G and G-C base pairs (Ciesiolka et al. 2017). Repeat expansion, correlated with the severity of disorders, increases the possibility of homodimer formation. Sufficiently long trinucleotide repeats can form foci in vivo through phase separation (Jain and Vale 2017). Homodimers are also formed by various ribozymes and riboswitches (Bou-Nader and Zhang 2020). Dimerization of RNAs is used in nanobiotechnology for the design and construction of RNA architectures through controlled self-assembly of modular RNA units (tectoRNAs) (Chworos et al. 2004; Guo 2010; Ishikawa et al. 2013; Geary et al. 2014; Grabow and Jaeger 2014; Tanaka et al. 2016). These observations suggest that transient and stable RNA homodimers play a role in a variety of physiological and pathological processes.

The last few years have seen the development of RNA proximity ligation methods to map cellular RNA–RNA interactions (Kudla et al. 2020). CLASH (Kudla et al. 2011), miR-CLIP (Imig et al. 2015), and hiCLIP (Sugimoto et al. 2015) use a protein bait to detect protein-associated RNA duplexes, whereas PARIS (Lu et al. 2016), LIGR-seq (Sharma et al. 2016), SPLASH (Aw et al. 2016), and COMRADES (Ziv et al. 2018) use a small molecule, psoralen, to cross-link interacting RNA strands. Proximity ligation methods have been commonly used to identify heterotypic interactions, such as interactions between snoRNA, miRNA, piRNA, or sRNA, and their respective targets (Kudla et al. 2011; Helwak et al. 2013; Grosswendt et al. 2014; Ramani et al. 2015). However, these methods also uncover many homotypic interactions, in which the two partners can be mapped to the same gene. Homotypic interactions, usually assumed to originate from the same RNA molecule, have been used to reveal the secondary structures of cellular RNAs (Kudla et al. 2011; Aw et al. 2016; Lu et al. 2016; Sharma et al. 2016) and structural dynamics of viral genomes (Ziv et al. 2018, 2020; Huber et al. 2019; Zhang et al. 2021b). However, homotypic interactions could also in principle represent binding between a pair of identical molecules forming an RNA homodimer. Here, we aimed to establish methods for the identification of homodimers in RNA proximity ligation data, benchmark experimental and computational protocols for mapping of homodimers, and globally profile the homodimerization of RNA in yeast and human cells and in Zika and SARS-CoV-2 viruses.

## Results

### Overlapping chimeras indicate intermolecular interactions

CLASH, PARIS, and other RNA proximity ligation methods rely on the ligation of interacting fragments of RNA, which are then detected as chimeric reads by high-throughput sequencing. We reasoned that chimeras that represent intra- and intermolecular interactions can be distinguished from each other by an analysis of sequence overlap between the arms of each chimera.

**Figure 1.**
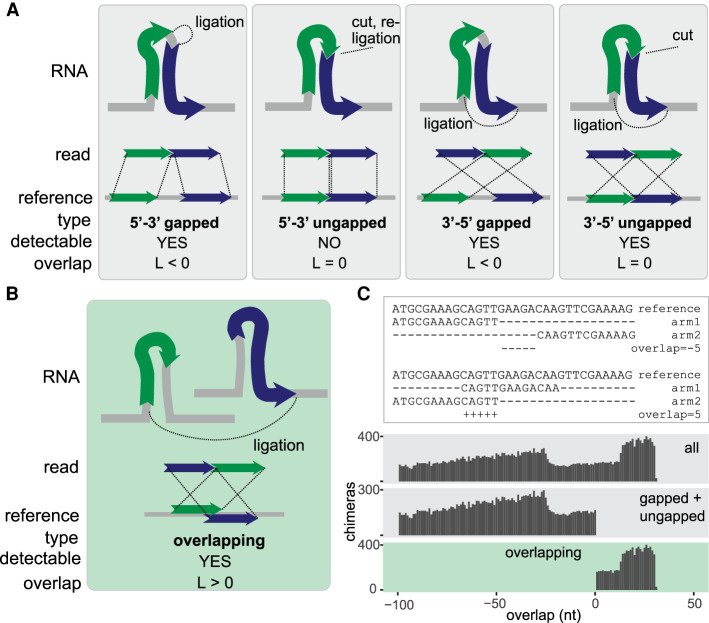
Classification of chimeric reads. (*A*) Types of nonoverlapping chimeras that may be formed by proximity ligation. The RNA fragments, shown as green and blue arrows, can originate from the same transcript (as shown in the figure) or from distinct transcripts (not shown). The dotted lines in the *upper* panel indicate ligation sites. (*B*) Diagram of an overlapping chimera. The RNA fragments originate from two distinct copies of a transcript but are mapped to the same region of the reference gene. (*C*, *top*) Examples of nonoverlapping and overlapping chimeric reads mapped to a reference gene. (*Bottom*) Distribution of the calculated overlap score (L) in all simulated chimeras, simulated nonoverlapping chimeras, and simulated overlapping chimeras.

When an RNA molecule that comprises an intramolecular interaction is subjected to proximity ligation, the RNA is fragmented into smaller pieces, which (by definition) originate from distinct parts of the RNA. When these fragments are ligated, sequenced, and mapped back to the reference, they should never be mapped to the same region of the RNA. The possible arrangements of the two fragments on the source RNA are shown in Figure 1A. Of these arrangements, the ungapped 5′-3′ chimera is indistinguishable from the source RNA sequence and cannot be identified as a chimera by a simple mapping approach. The other arrangements—gapped 5′-3′, ungapped 3′-5′, and gapped 3′-5′—are all feasible and are commonly detected in proximity ligation experiments.

In contrast, when an intermolecular interaction exists between two copies of the same RNA molecule, the two interacting fragments may or may not originate from the same part of the RNA. When these fragments are mapped to the source RNA sequence, they can be found in any of the arrangements shown in Figure 1A and in an additional overlapping arrangement (Fig. 1B). Thus, gapped and ungapped chimeras can result from intra- and intermolecular interactions, but overlapping chimeras are diagnostic of intermolecular interactions.

In the following sections, we discuss the suitability of bioinformatic methods to detect the relevant types of chimeric reads; the types of chimeras we identify in various RNA proximity ligation experiments; and the possible origin and interpretation of the interactions we detect.

### Detection of overlapping chimeras in simulated sequencing data

To identify chimeras, we used the hyb pipeline (Travis et al. 2014). Hyb maps reads against a reference sequence database with one of several tools (BLAST, Bowtie 2, or BLAT) (Altschul et al. 1990; Kent 2002; Langmead and Salzberg 2012) and detects chimeric reads with two separate local matches in the database. To test whether hyb is suitable for detection of gapped, ungapped, and overlapping chimeras, we assembled a test data set with simulated chimeras by concatenating all possible pairs of 30-nt substrings from an arbitrary 228-nt RNA sequence. Using either BLAST or Bowtie 2 as the mapping engine, hyb correctly identified the majority of sequences as chimeric. A subset of 5′-3′ gapped chimeras and overlapping chimeras were not called by the algorithm (Supplemental Fig. S1). Inspection of the BLAST and Bowtie 2 outputs showed that these chimeras were interpreted by the mapping programs as nonchimeric reads with internal deletions or insertions. We also called chimeras with STAR, a general-purpose mapping tool that has been used in some RNA proximity ligation studies (Dobin et al. 2013). Although the results were comparable with hyb, STAR missed most 5′-3′ chimeras and a subset of 3′-5′ chimeras (Supplemental Fig. S1). Both hyb and STAR commonly misidentified the position of the ligation junction between the two arms of the chimera by 1–3 nt, but this did not affect the identification of overlapping chimeras, which typically relies on the mapped position of nonligated ends of reads (Fig. 1B). Using an alternative test data set with more than 1 million simulated nonchimeric reads and more than 1 million chimeric reads (Methods), we found 99.9% specificity and 95% sensitivity in the detection of nonoverlapping chimeras, and 100.0% specificity and 69% sensitivity in the detection of overlapping chimeras, where most false negatives were chimeras with very short overlaps.

We then quantified the degree of overlap between arms of chimeric reads using an overlap metric L defined for chimeras where both arms are mapped to the same reference transcript as follows:

$$L=1+\min(e_{1},\;e_{2})-\max(s_{1},\;s_{2}),$$

in which s_1_ and s_2_ are start mapping coordinates of arms 1 and 2 of the chimera on the reference transcript, and e_1_ and e_2_ are end coordinates of arms 1 and 2. L is positive for overlapping chimeras, null for ungapped 3′-5′ chimeras, and negative for gapped 5′-3′ or 3′-5′ chimeras. In the test data set, L was positive for simulated overlapping reads and negative for simulated nonoverlapping reads (Fig. 1C), as expected. These results show that our methods are appropriate for the identification of overlapping chimeras in RNA proximity ligation data.

### Overlapping chimeras in RNA proximity ligation data

We analyzed representative RNA proximity ligation data sets generated by several experimental protocols (Helwak et al. 2013; Ramani et al. 2015; Aw et al. 2016; Lu et al. 2016; Sharma et al. 2016; Waters et al. 2016; Li et al. 2018; Ziv et al. 2018, 2020; Huber et al. 2019; Cai et al. 2020; Methods). The protocols differ, among other ways, in the method used to stabilize RNA–RNA interactions. CLASH uses UV-protein cross-linking, with only one RNA strand expected to be covalently linked to a protein and the other bound by complementarity. SPLASH, PARIS, and COMRADES use psoralen cross-linking, whereas RIC-Seq is based on protein-dependent formaldehyde cross-linking, and RPL omits the cross-linking step altogether.

**Figure 2.**
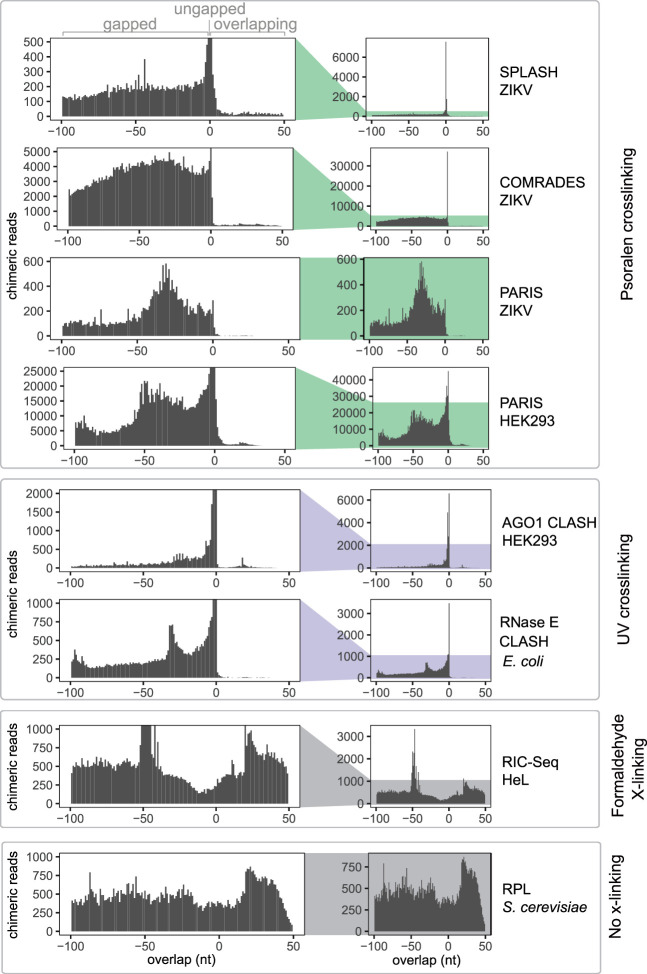
UV and psoralen cross-linking predominantly generate gapped and ungapped chimeras. The distribution of overlap scores (L) across proximity ligation data sets: SPLASH ZIKV (Zika virus) (Huber et al. 2019), COMRADES ZIKV (Ziv et al. 2018), PARIS ZIKV (Li et al. 2018), PARIS HEK293 (human) (Lu et al. 2016), AGO1 CLASH HEK293 (Helwak et al. 2013), RNase E CLASH (*Escherichia coli*) (Waters et al. 2016), RIC-Seq HeLa (human) (Cai et al. 2020), and RPL (*Saccharomyces cerevisiae*) (Ramani et al. 2015; Methods).

We focused on homotypic chimeras, that is, those in which both arms are mapped to the same transcript. Among homotypic chimeras, we detected gapped, ungapped, and overlapping chimeras in all data sets, but the relative proportions of these three types varied greatly between data sets (Fig. 2). Methods that use UV and psoralen cross-linking to recover direct RNA:RNA interactions yielded large numbers of gapped and ungapped, but few overlapping chimeras. For example, out of 4.1 million chimeras we detected in the PARIS HEK293 data set, 3.2 million were homotypic chimeras, but only 42,000 were overlapping homotypic chimeras, indicative of RNA homodimerization (Supplemental Fig. S2). Although gapped chimeras could originate from inter- or intramolecular interactions, the near absence of overlapping chimeras suggests that homomeric intermolecular interactions are rare in these data sets. In contrast, RPL and RIC-Seq recovered large numbers of overlapping chimeras. Both RPL and RIC-Seq can plausibly recover indirect interactions: RIC-Seq was specifically designed to detect indirect contacts through protein formaldehyde cross-linking, and RPL might allow for reassociation of RNA:RNA complexes during chemical processing in situ, owing to the absence of a covalent linkage between RNA strands. These results suggest that RNA homodimerization mediated by direct RNA–RNA base-pairing is uncommon in vivo. The results also show that RNA duplexes are generally stable during library preparation, at least in the CLASH, SPLASH, PARIS, and COMRADES methods, because random reassociation of duplexes would lead to the formation of similar proportions of gapped and overlapping chimeras.

An intriguing pattern is the peak at overlap = 0 in Figure 2, indicating the preferential recovery of 3′-5′ ungapped chimeras relative to gapped and overlapping chimeras (as discussed above, 5′-3′ ungapped chimeras cannot be detected with our methods). We propose that ungapped chimeras typically arise from local RNA stem–loop structures, which are subject to three endonucleolytic cleavages, followed by ligation of the distal ends to each other, whereas gapped chimeras could be created either by four independent endonucleolytic events, or by a combination of three endonucleolytic cuts combined with exonucleolytic trimming of RNA ends. Although enrichment of ungapped chimeras can be readily explained for intramolecular interactions, it is difficult to imagine a mechanism that could enrich ungapped chimeras for intermolecular interactions. These results reinforce our conclusion that stable RNA homodimers are rarely formed in vivo.

We also observed an overrepresentation of overlapping chimeras with very short overlaps (0 < L < 5) in all UV and psoralen data sets (Fig. 2; Supplemental Fig. S2). We speculate that most such chimeras are derived from the same type of interaction that gives rise to 3′-5′ ungapped chimeras, but the apparent overlap is caused by mapping errors. Although we could not detect similar artifacts in our simulated benchmarking data, the artifacts could arise in experimental data because of sequencing errors or adapter mutations. We thus conservatively exclude chimeras with L < 5 from consideration in the calling of homodimers.

### Homodimerization of human and yeast RNAs

**Figure 3.**
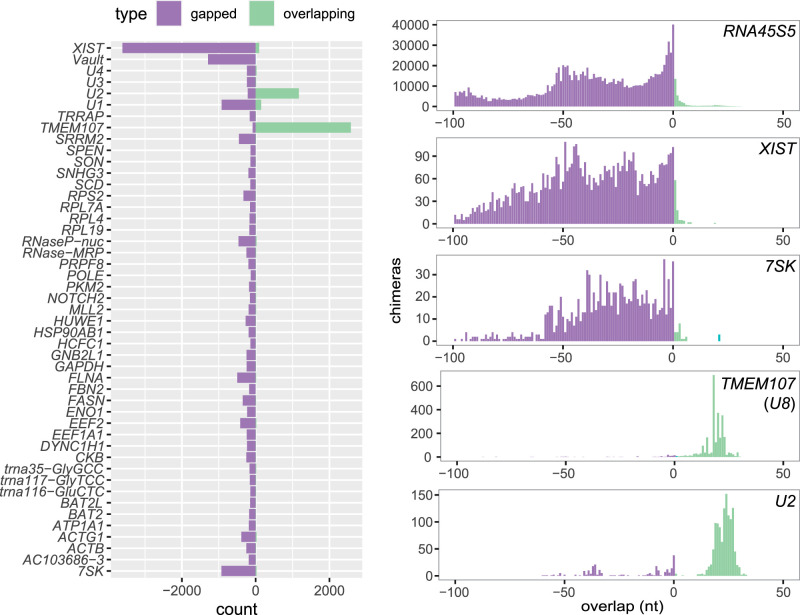
Distribution of overlap scores in individual genes in PARIS experiment. Counts of gapped and ungapped chimeras (purple) and overlapping chimeras (green) in individual genes in PARIS data from human HEK293 cells (Lu et al. 2016) (*left*), and distribution of the overlap value L in selected genes.

Although few RNA homodimers were found in UV and psoralen cross-linking experiments, we hypothesized that homodimers might be limited to a specific subset of RNAs. To investigate this possibility, we analyzed chimeras detected in individual genes in transcriptome-wide PARIS data from HEK293 cells. To increase the stringency of our analysis, we filtered the data to remove likely mapping errors, chimeras with thermodynamically unstable interactions, homopolymers, and chimeras with very short overlaps (<5 nt) (Supplemental Fig. S2). After filtering, gapped chimeras were more common overall, but 84 genes contained overlapping chimeras, including 17 genes that were significantly enriched in overlapping chimeras (Fisher's exact test with Benjamini–Hochberg correction, *P* < 0.05) (Supplemental Fig. S3; Supplemental Data Set S1). The most highly enriched transcript was mRNA *TMEM107*, which contained 100 times more overlapping than gapped chimeras (Fig. 3).

**Figure 4.**
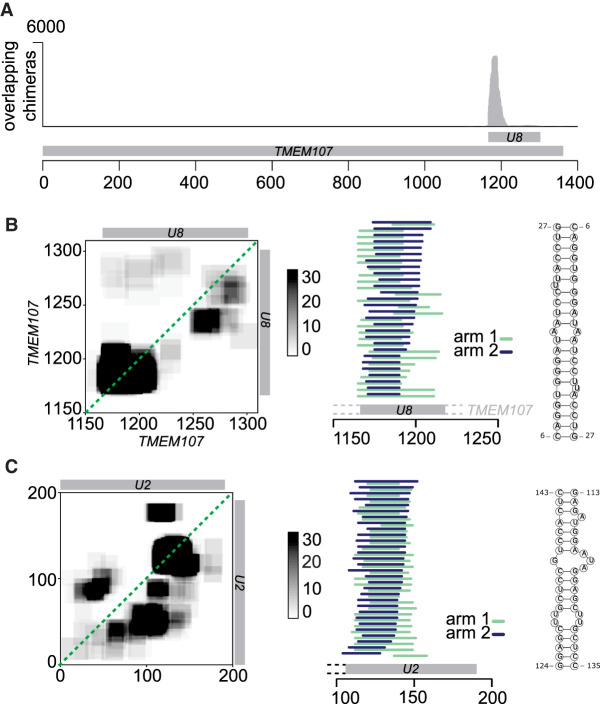
Homodimerization of *U8* snoRNA and *U2* snRNA. (*A*) Distribution of overlapping chimeras along the *TMEM107* gene in PARIS (HEK293 cells) (Lu et al. 2016). All overlapping chimeras coincide with the position of *U8* snoRNA in the 3′ UTR of *TMEM107* transcript. (*B*, *left*) Coverage map of *U8*:*U8* chimeras. Numbers on the *x-* and *y*-axes indicate positions in the *TMEM107* transcript. The position of mature *U8* snoRNA is indicated by the gray bars. (*Middle*) Mapping positions of both arms of chimeras along *TMEM107*. Some chimeras extend beyond the 5′ end of mature *U8* (gray bar), indicating that they originate from the *U8* precursor molecule. (*Right*) Predicted base-pairing of *U8*:*U8* homdimer. (*C*) As above, analysis of *U2*:*U2* chimeras. Coordinates indicate positions along the *U2* transcript.

*TMEM107* contains a small nucleolar RNA (snoRNA), *U8*, in its 3′ untranslated region, and almost all *TMEM107*:*TMEM107* chimeras mapped to that region, suggesting that these chimeras represent *U8*:*U8* interactions. The chimeras were concentrated around the 5′ end of *U8* (Fig. 4A,B), and RNA folding prediction showed extended self-complementarity in this part of the transcript, consistent with homodimerization with a predicted free energy of −21 kcal/mol. The same *U8*:*U8* interaction was identified in CLASH data, and in an independent analysis of a new PARIS2 data set (Zhang et al. 2021a, 2022). Previous studies showed that the 5′ region of *U8* may base pair with pre-ribosomal RNA (Peculis 1997; Zhang et al. 2021a) and with the 3′ end of a 3′-extended precursor of *U8* (Badrock et al. 2020). Because homodimerization seems incompatible with these interactions, it might represent an immature form of *U8* or play a role in the regulation of *U8* function. This is potentially important for the pathogenesis of LCC, a neurological disease caused by loss-of-function mutations in *U8* (Jenkinson et al. 2016; Badrock et al. 2020). Some overlapping chimeras comprised a 5′-extended form of *U8*, indicating that the homodimers may be formed during snoRNA maturation. We have not found any interactions involving other regions of the *TMEM107* transcript (Supplemental Fig. S3), nor have we found homodimer enrichment in other mRNAs from the *TMEM* family.

In addition to *U8*:*U8* interactions, analysis of PARIS data showed enrichment of homodimers in *U1* and *U2* snRNA (Figs. 3, 4C; Supplemental Fig. S4). *U2* snRNA contained 20 times as many overlapping as gapped chimeras. Regions involved in homodimeric interactions in *U1* and *U2* are limited to a particular fragment of the RNA, whereas other types of interactions can be found along the transcript (Supplemental Fig. S3). Most overlapping chimeras in *U2* included the sequence of stem–loop III, downstream from the Sm binding site, suggesting that in a fraction of *U2* molecules found in the cell, stem–loop III is unfolded and forms homomeric intermolecular interactions (Fig. 4C). Out of the two major isoforms of *U2*, *U2-1* shows more efficient deposition of the Sm ring and incorporation into snRNP complexes than *U2-2* (Kosmyna et al. 2020), and we hypothesized that failure of assembly into an snRNP complex might be associated with *U2*:*U2* dimerization. However, analysis of the exact sequences of *U2*:*U2* overlapping chimeras showed that out of 223 reads that could be assigned to specific *U2* isoforms, 162 were of the *U2-1*:*U2-1* type, and 59 were *U2-1*:*U2-2* chimeras, suggesting that both U2 isoforms may form homo- and heteromeric intermolecular interactions. *U2* homodimers were also found in CLASH, SPLASH (this study), and PARIS2 (Zhang et al. 2022).

Altogether, across the five studies we analyzed (AGO1 CLASH, PARIS, Zika COMRADES, Zika SPLASH, and human SPLASH), we found 50 transcripts with homodimers found across two or more studies (Supplemental Fig. S3). These transcripts include ribosomal RNA, *U1* and *U2* snRNA, *U3* and *U8* snoRNA, 4 tRNAs, and 36 mRNAs. We then compared specific homodimerization events detected by different proximity ligation methods. PARIS and COMRADES showed the largest fractions of homotypic chimeras, most of which were nonoverlapping and likely represented intramolecular interactions. Across all RNA biotypes, rRNAs formed most homodimers, particularly in PARIS and SPLASH, but such homodimers were not statistically enriched, when compared to intramolecular interactions. tRNAs were enriched for homodimers in AGO1 CLASH and SPLASH (Supplemental Figs. S3, S5). tRNA-derived small RNAs (tsRNAs), including tRNA-derived fragments (tRFs) and tRNA halves (tiRNAs), are small regulatory RNAs processed from mature tRNAs or precursor tRNAs (Xie et al. 2020). *tX(XXX)D*, a yeast tRNA similar to serine tRNAs (Chan and Lowe 2009) formed a homodimer through a 12 base pair long stem in SPLASH data (Supplemental Fig. S6). The tRNA homodimers detected by AGO1 CLASH in human cells (Supplemental Fig. S6) may indicate a miRNA-tRNA network resulting in competition for binding sites and availability for gene silencing, as reported previously (Shigematsu and Kirino 2015).

The largest ratio of overlapping to homotypic chimeras was recovered by RIC-Seq (7%). RIC-Seq also recovered the highest number of genes with overlapping chimeras (more than a thousand), 17 of which were significantly enriched for overlapping chimeras (Supplemental Fig. S3). As discussed above, the overlapping chimeras found by RIC-Seq probably represent indirect interactions rather than RNA homodimers. RIC-Seq showed significant enrichment of overlapping chimeras in some mitochondrial mRNAs, with *CO1*, *ND2*, and *ND4* containing the highest numbers of overlaps (Supplemental Figs. S3, S7). Bidirectional transcription of mitochondrial RNA is known to result in hybridization of complementary strands (Dhir et al. 2018; Kim et al. 2018), but in the RIC-Seq data, both partners come from the same strand, suggesting that they represent a distinct type of interaction. The mitochondrial mRNA:mRNA chimeras showed low thermodynamic stability and short regions of complementarity (2–8 base pairs), suggesting that these chimeras represent indirect interactions facilitated by the high local concentrations of transcripts in mitochondria. COMRADES, PARIS, and SPLASH also detected homodimers among mitochondrial transcripts (Supplemental Fig. S4). *YLR154W-E*, a possible ncRNA from yeast with a strong enrichment in overlapping chimeras in the RPL data, can be predicted to dimerize through an extended stem structure (Supplemental Fig. S7).

### The *U8* homodimerization domain plays an important role in vivo

To study the function of homodimers in more detail, we focused on the *U8*:*U8* interaction, the most abundant homodimer in our analysis of the PARIS data. We took advantage of an experimental system in which *U8-3*^−/−^ zebrafish embryos are injected with human *U8* precursor RNA (pre-*U8*) to analyze the functional consequences of human *U8* mutations (Badrock et al. 2020). Previous experiments showed abnormal yolk sac and brain development in *U8-3*^−/−^ embryos, and that these phenotypes were complemented by injection of wild-type human pre-*U8*, but not by injection of known disease mutants of *U8* (Badrock et al. 2020). Thus, the zebrafish model can identify loss-of-function mutations in human *U8*.

**Figure 5.**
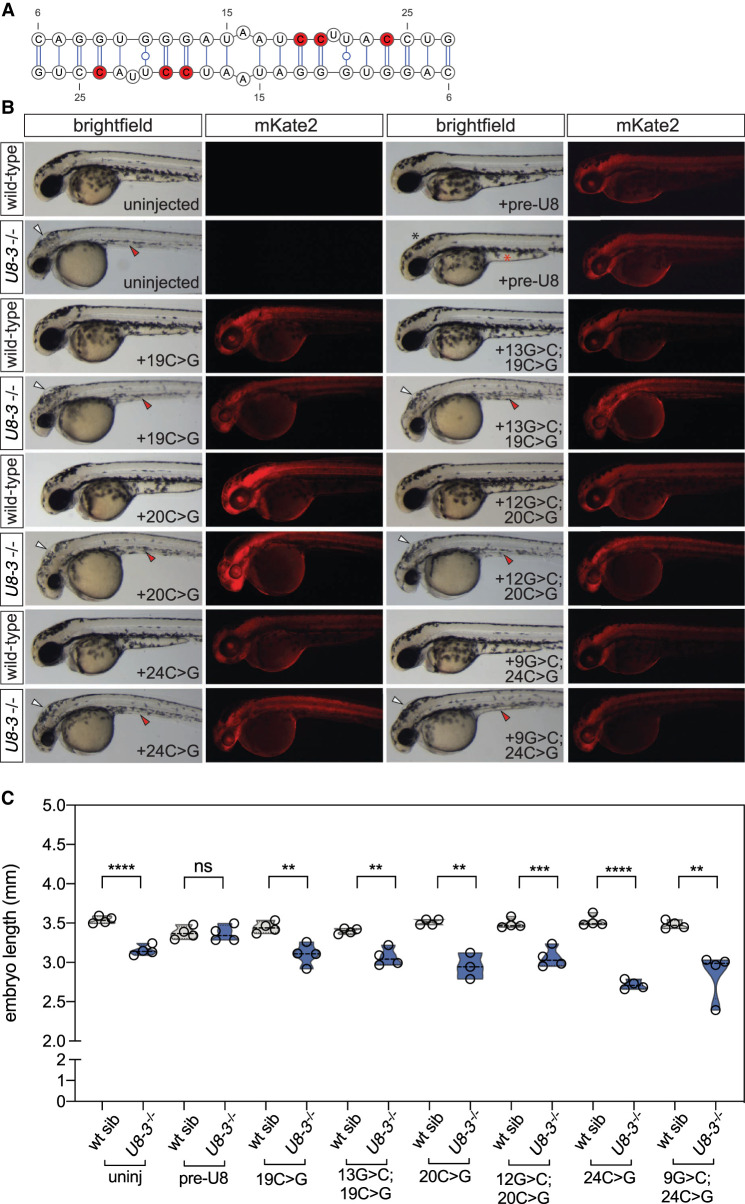
Functional analysis of *U8* homodimerization domain. (*A*) *U8*:*U8* homodimer with positions of selected destabilizing mutations indicated in red. (*B*) Representative brightfield and fluorescent images of the indicated genotype and exogenous precursor *U8* snoRNA variants taken at 48 h post-fertilization. mRNA encoding the mKate2 fluorescent protein was coinjected to show successful uptake of the injected solution into the embryo. White arrowheads denote hydrocephaly, red arrowheads denote aberrant yolk extension, whereas a black asterisk denotes an absence of hydrocephaly and a red asterisk denotes rescued yolk morphology. (*C*) Quantitation of embryo length for the genotypes and introduced precursor *U8* snoRNA variants indicated. (uninj) Uninjected. n = 3–4 embryos per genotype. Black dashed line indicates median value; all data points are shown.

We first asked if mutations predicted to affect homodimer formation disrupt *U8* function in zebrafish. We selected three candidate mutations: 19C > G, 20C > G, and 24C > G, which are expected to strongly disrupt *U8* homodimerization, but have little or no effect on the predicted interactions of *U8* with the preribosome or on any other known domain or function of *U8* (Fig. 5A; Supplemental Fig. S8). We found that all three mutants fail to complement developmental phenotypes observed in the *U8-3*^−/−^ embryos (Fig. 5B,C). We also tested candidate mutations 20C > T and 24C > T, which are predicted to disrupt the *U8* homodimer, and that had been found in patients suffering from LCC, a neurodegerative disease caused by the loss of *U8* function. Again, these mutants fail to complement the zebrafish phenotype (Supplemental Fig. S8). Furthermore, mutations in the homodimerization domain disrupt the formation of slowly migrating conformers by in vitro transcribed *U8* RNA in native polyacrylamide gel electrophoresis (Supplemental Fig. S9). Taken together, these results suggest that the *U8* homodimerization domain we discovered plays an important biological role that is conserved across vertebrates.

Next, we attempted to rescue homodimerization mutants by compensatory mutations. None of the five double mutants we tested rescued the phenotype of zebrafish embryos (Fig. 5B,C; Supplemental Fig. S8). We postulate that compensatory mutations disrupt other important aspects of *U8* function, and thus they do not complement function, although they may rescue dimerization. This interpretation is supported by the predicted effect of our compensatory mutations on the *U8*:28S pre-rRNA interaction (Supplemental Fig. S8). Further biochemical studies are required to dissect the functions of the *U8* homodimerization domain and its mutated variants. Altogether, our experiments are consistent with the hypothesis that *U8*:*U8* and *U8*:28S pre-rRNA interactions are essential for *U8* function in vivo.

### Homodimerization of virus RNA

**Figure 6.**
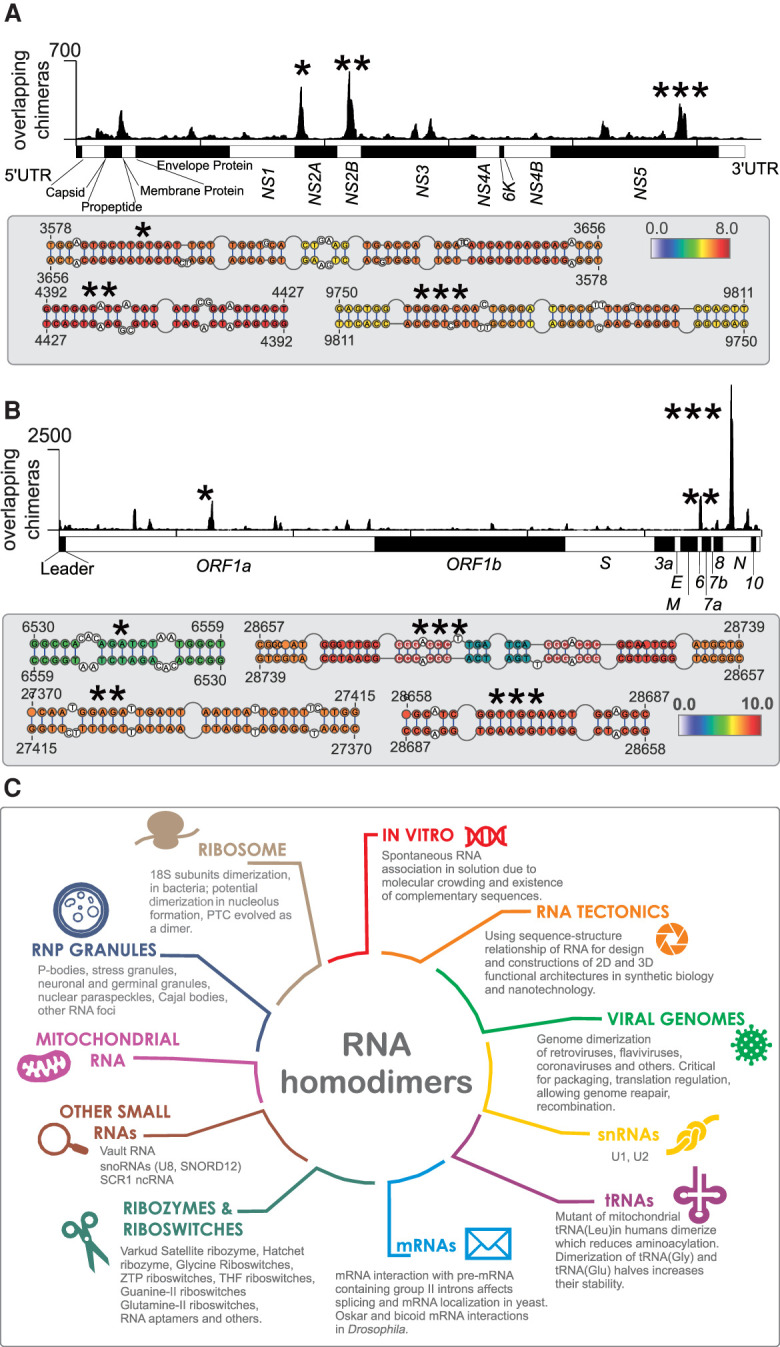
Homodimers of viral genomes. (*A*, *top*) Coverage of overlapping chimeras in Zika virus identified by COMRADES (Ziv et al. 2018). The regions with the largest coverage of homodimers are indicated by asterisks. (*Bottom*) Predicted secondary structures of regions with high coverage of overlapping chimeras. The colors indicate the COMRADES score, that is, the log_2_ of the number of chimeric reads supporting each base pair. (*B*) Coverage of overlapping chimeras along the SARS-CoV-2 genome identified by COMRADES (Ziv et al. 2020). The top homodimeric regions and their predicted structures are shown, as described above. (*C*) Known types of RNA homodimers.

We next turned to COMRADES data from cells that have been infected with SARS-CoV-2 and Zika viruses, to detect possible homodimers of virus RNA. Although Zika RNA is not known to homodimerize, dimerization is an essential step in the packaging of some viruses, including HIV, whereas dimerization of SARS-CoV RNA was suggested to play a role in translational frameshifting (Ishimaru et al. 2013). To detect dimers of virus RNA, we analyzed the coverage of overlapping chimeras along viral genomes. Unlike gapped chimeras, which covered the Zika genome relatively evenly, overlapping chimeras were strongly enriched in several positions within the *NS2A*, *NS2B*, and *NS5* coding sequences of the Zika virus, indicating possible dimerization sites (Fig. 6A). RNA folding prediction showed regions of self-complementarity in the interaction sites, including a pair of uninterrupted 11-bp duplexes in the (3578–3656):(3578–3656) region in the *NS2A* gene. However, folding energy alone was not enough to predict dimerization sites, as evidenced by the weak negative correlation between the count of overlapping chimeras in a genomic window and the predicted strength of homodimeric interaction in that window (Pearson R = −0.17, *P* = 3 × 10^−8^).

We also detected dimerization events in the SARS-CoV-2 genome, with the largest peak in the nucleoprotein (*N*) gene, and additional peaks in the *N*, *Orf6*, and *Orf1a* coding sequences (Fig. 6B). The region with the largest coverage of overlapping chimeras was 200 nt long (coordinates 28,610–28,810) and the resolution was insufficient to indicate the exact base-pairing, but RNA folding analysis showed several high-scoring interactions, including a 10-nt duplex formed by the palindromic sequence, GGTTGCAACT. Although a previous NMR study detected a functionally important dimerization site near the frameshifting element of the SARS-CoV virus (Ishimaru et al. 2013), our analysis shows no obvious enrichment of overlapping chimeras in the homologous region of SARS-CoV-2.

## Discussion

Although homo-oligomerization is common in proteins, few RNA homo-oligomers have been described in vivo. This is somewhat surprising, given that RNA molecules readily homodimerize in vitro, to the point that special procedures have to be used to isolate monomeric forms of certain RNAs for structural studies (Zhang and Ferre-D'Amare 2014; Bou-Nader and Zhang 2020). The paucity of in vivo homo-oligomers might be explained by the folding of RNAs and by their association with protein complexes, which reduce the propensity for *trans*-RNA–RNA interactions. Alternatively, the apparent lack of in vivo homodimers might simply reflect the lack of systematic studies of dimerization. Here, by analyzing the relative proportions of gapped, ungapped, and overlapping chimeric reads in RNA proximity ligation experiments, we find that homodimerization mediated by direct RNA base-pairing is indeed rare in vivo. However, we find that certain human RNAs and some regions of the RNA genomes of the Zika and SARS-CoV-2 viruses are enriched for in vivo homodimers.

Out of thousands of RNAs we examined, only a handful show clear evidence of dimerization. The propensity to dimerize is necessarily influenced by the primary sequence of the RNA: for example, palindromic sequences or CAG repeats might be prone to form intermolecular interactions. A recent review of RNA homodimer structures detected in viruses, ribozymes, and riboswitches identified preferences for certain sequence and structural arrangements, such as palindromes, complementary strand swapping, and kissing-loop interactions (Bou-Nader and Zhang 2020). Indeed, in the present study, palindromic sequences were found in several RNA homodimers. Homodimerization is also likely to be influenced by folding kinetics, association with proteins and other RNAs, subcellular localization, and local concentration of RNA and metal ions. RNA molecules that fold cotranscriptionally into stable secondary structures are unlikely to form extended duplexes with other RNAs (Yu et al. 2021), whereas molecules that are unfolded by helicases, or located in granules with high local concentrations of a given RNA, might be more likely to form transient or stable oligomers. Copies of RNA molecules located in close proximity may initially interact with a few nucleotides, followed by destabilization of local structure and nucleation of longer interactions (Ganser et al. 2019).

Are the RNA homodimers detected by proximity ligation biologically relevant, or are they experimental artifacts? We argue that nonspecific dimerization and oligomerization of RNA during library preparation, if present, should lead to the formation of many overlapping chimeras, distributed across a large variety of RNAs. Indeed, this is what we observe in the RPL and RIC-Seq data sets. RPL is performed without cross-linking, whereas RIC-Seq involves formaldehyde cross-linking. As a result, overlapping chimeras detected by these methods likely indicate local transcript proximity rather than direct base-pairing, although it is also possible that a fraction of overlapping chimeras arises during the library preparation step.

In contrast, techniques that rely on UV or psoralen cross-linking—CLASH, SPLASH, PARIS, and COMRADES—are expected to detect RNA–RNA contacts mediated by direct base-pairing. We observed that these methods generate few overlapping chimeras, but these chimeras are strongly enriched in a small subset of RNAs, suggestive of bona fide interactions. Alternatively, overlapping reads might theoretically arise through reverse transcription of an endogenous circular RNA (circRNA), or of an artificial circRNA created in vitro by ligation, producing a concatemeric cDNA. However, the low abundance of circRNAs and low efficiency of RNA ligases makes such events unlikely. We also note that proximity ligation can only identify a subset of possible RNA homodimers, namely, those where both RNAs interact via the same part of their sequence, or via two regions that are close enough in the primary sequence to detect overlaps in chimeric reads. Although many known RNA homodimers are of this type (e.g., the DIS kissing loop interaction in HIV, the SL2-SL2 interaction in Moloney murine sarcoma virus [MoMuSV], or the dimerization of *Oskar* RNA via its 3′ UTR in *Drosophila* embryos) (Berkhout and van Wamel 1996; Kim and Tinoco 2000; Jambor et al. 2011), interactions mediated via distant fragments of RNA would not be detectable by proximity ligation.

Homodimerization has now been reported for most major biotypes of RNA, and known roles of homodimers include the packaging of viral genomes, assembly of membraneless organelles, regulation of RNA localization (Fig. 6C). Given its dependence on the local concentration of RNA, dimerization might play a role in RNA quorum sensing—a process analogous to that used by bacteria and viruses to coordinate their behavior in response to the local population density. Nevertheless, many RNA homodimers do not have a known biological function, and indeed might be detrimental. Stretches of dsRNA are known to trigger antiviral immunity through PKR and other cellular factors (Hull and Bevilacqua 2016), and some types of homodimers might be misidentified as foreign RNA. RNA multimerization has also been associated with general cellular stress (Van Treeck and Parker 2018; Van Treeck et al. 2018). In RNA proximity ligation methods, the use of psoralen, formaldehyde, and UV light is a stress factor that might contribute to RNA multimerization. In any case, further functional studies are required to elucidate the roles of the wide variety of RNA homodimers that can be detected in our cells.

## Methods

### Benchmarking chimera detection on test data

To benchmark methods for detection of overlapping chimeras, we assembled a test data set using an arbitrary RNA sequence (nucleotides 1–228 of *S. cerevisiae RDN37* gene: NCBI GenBank database (https://www.ncbi.nlm.nih.gov/genbank/) Sequence ID: CP026300.1, range 448,071–448,298, minus strand). We generated all 30-nt substrings of the reference sequence and concatenated all possible pairs of substrings, which yielded 10,871 overlapping chimeras and 28,730 nonoverlapping chimeras.

We then called chimeras in the overlapping and nonoverlapping data sets using hyb and STAR, using the following commands:

hyb (Bowtie 2 mapping):

hyb analyse in = input.fasta db = RDN37 format = comp eval = 0.001

hyb (BLAST mapping):

hyb analyse in = input.fasta db = RDN37 format = comp align = blastall eval = 0.001

STAR:

STAR ‐‐genomeDir . ‐‐readFilesIn input.fasta ‐‐outFileNamePrefix 06 ‐‐outReadsUnmapped Fastx ‐‐outFilterMismatchNoverLmax 0.05 ‐‐outFilterMatchNmin 16 ‐‐outFilterScoreMinOverLread 0 ‐‐outFilterMatchNminOverLread 0 ‐‐clip3pAdapterMMp 0.1 ‐‐chimSegmentMin 15 ‐‐scoreGapNoncan -4 ‐‐scoreGapATAC -4 ‐‐chimJunctionOverhangMin 15

We used the ua.hyb files from hyb and Chimeric.out.junction files from STAR for downstream analysis. To generate the coverage heatmaps of chimeras detected in the test data set, we extracted the coordinates of chimera junctions and plotted them using Java TreeView (Saldanha 2004).

As a second test data set, we used more than 1 million simulated chimeric reads and more than 1 million nonchimeric reads from the *S. cerevisiae* preribosomal RNA (*RDN37*; 6858 nt in length). The lengths of chimeric arms ranged from 20 to 40 nt, to approximate the sizes of chimeric fragments found by CLASH, PARIS, and related methods. We mapped these simulated reads against the entire yeast transcriptome, using hyb with default settings. To estimate sensitivity and specificity for chimera detection, we used L ≥ 5 (see next section) as the cutoff for calling overlapping chimeras.

### Calculation of chimera overlaps

To quantify overlap between arms of chimeric reads, we defined the overlap metric, L, as

$$L=1+\min(e_{1},\;e_{2})-\max(s_{1},\;s_{2}),$$

where L is defined for chimeras in which both arms are mapped to the same reference transcript, or same chromosome in case of mapping to a genome reference. e_1_ represents the end mapping coordinate of the left arm of the chimera (arm 1); e_2_ represents the end mapping coordinate of the right arm of the chimera (arm 2); s_1_ and s_2_ represent the start mapping coordinates of the respective arms. Calculation of L was implemented as a custom awk script, taking the ua.hyb files produced by the hyb pipeline as inputs.

### RNA proximity ligation data

We downloaded the data from the following and including NCBI (https://www.ncbi.nlm.nih.gov/) and ArrayExpress (https://www.ebi.ac.uk/arrayexpress/) accession numbers: *Escherichia coli* RNase E CLASH: GSE77463 (Waters et al. 2016); human AGO1 CLASH: GSE50452 (Helwak et al. 2013); human SPLASH: SRR3404931 (Aw et al. 2016); Zika SPLASH: SRR6252011 (Huber et al. 2019); Zika COMRADES: E-MTAB-6427 (Ziv et al. 2018); human LIGR-Seq: SRR3361013 (Sharma et al. 2016); human PARIS: SRR2814765 (Lu et al. 2016); Zika PARIS: PRJEB28648 (Li et al. 2018); human RIC-Seq: SRR8632820 (Cai et al. 2020); *Saccharomyces cerevisiae* RPL: SRR2048219 (Ramani et al. 2015); and SARS-CoV-2 COMRADES: GSM4676632 (Ziv et al. 2020).

Sequencing data were downloaded in FASTQ format (except for the SARS-CoV-2 data set, from which hyb output files were downloaded). Chimeric reads were called and annotated with the hyb package (Travis et al. 2014) with default settings, using the appropriate transcriptome database (Helwak et al. 2013; Waters et al. 2016; Ziv et al. 2018), as described in Supplemental Data Set S2.

Overlap statistics across experimental data sets were visualized in R (R Core Team 2017) using the ggplot2 and ggforce libraries (facet_zoom function). To identify genes enriched in overlapping chimeras, we filtered hyb outputs to remove possible mapping errors (any reads with nucleotide repeats of length 15 or more, and chimeras where either arm had a mapping e-value greater than 0.001); we also removed chimeras with predicted interaction energy weaker than −5 kcal/mol. Because very short overlaps might represent mapping or sequencing errors, we conservatively called chimeras with overlap score L ≥ 5 as overlapping, and chimeras with −50 < L < 0 as nonoverlapping. We then assembled a contingency table with counts of overlapping and nonoverlapping chimeras for the focal gene and for all other genes, and we used a Fisher's exact test with Benjamini–Hochberg multiple testing correction to identify genes with significant enrichment of overlapping chimeras.

#### Zebrafish U8-3 mutant rescue experiments

Human *U8* RNA variants were in vitro transcribed from DNA templates containing a T7 consensus sequence as described (Badrock et al. 2020). pCS2+-mKate2 was linearized with NotI, and mRNA transcribed from the DNA template using the mMESSAGE mMACHINE SP6 transcription kit (Thermo Fisher Scientific) according to the manufacturer's instructions. Microinjections of 2 nL of solution containing 500 pg of a *U8* variant and 100 pg *mKate2* mRNA were microinjected into the yolk of one-cell stage zebrafish embryos through use of the PicoSpritzer III (Parker Instruments) apparatus. Where *U8* variants were found to rescue the morphology of *U8-3* mutant zebrafish, genotyping was performed as described in Badrock et al. (2020) to confirm the genotype of the assessed embryos.

#### Imaging and embryo measurement

Zebrafish embryos were anesthetized using MS-222 (Sigma-Aldrich), embedded in 3% Methyl cellulose (M0387), and imaged on an MZFLIII fluorescent stereomicroscope (Leica) with a MicroPublisher 3.3 RTV camera, using Micro-Manager 1.4.23 software. Embryo length was quantified using images taken at 1× magnification with a 1-mm scale bar as a reference point. Embryo length was measured in Microsoft Powerpoint (Microsoft) by drawing a line from head to tail.

#### Statistics and reproducibility

Statistical analyses were performed using GraphPad Prism 8.0. Results are presented as violin plots, with bold dashed line representing the median value. All data points are shown. For all analyses, *P* < 0.05 was considered statistically significant (using a student *t*-test). Statistical methods were not used to predetermine sample size. Experiments were not randomized. The investigators were not blinded to allocation during experiments and outcome assessment.

Primers unique to this study were mutated nucleotide lowercase, T7 sequence red, bolded Gs to ensure accurate transcription of entire *U8* snoRNA. Common reverse primer and primers used to transcribe the wild-type precursor form of *U8*, and primers used to genotype *U8-3* mutant zebrafish are reported in Badrock et al. (2020).

 8G > A sense: TAATACGACTCACTATA**GGGG**ATCGTCAaGTGGGATAATCCTTACCTG
 19G > C sense: TAATACGACTCACTATA**GGGG**ATCGTCAGGTGGGATAATgCTTACCTGTTCCTCCTC
 13C_19G sense: TAATACGACTCACTATA**GGGG**ATCGTCAGGTGGcATAATgCTTACCTGTTCCTCCTC
 20C > G sense: TAATACGACTCACTATA**GGGG**ATCGTCAGGTGGGATAATCgTTACCTGTTCCTCCTC
 12G > C_20C > G sense: TAATACGACTCACTATA**GGGG**ATCGTCAGGTGcGATAATCgTTACCTGTTCCTCCTC
 20C > T sense: TAATACGACTCACTATA**GGGG**ATCGTCAGGTGGGATAATCtTTACCTGTTCCTCCTC
 12G > A_20C > T sense: TAATACGACTCACTATA**GGGG**ATCGTCAGGTGaGATAATCtTTACCTGTTCCTCCTC
 24C > G sense: TAATACGACTCACTATA**GGGG**ATCGTCAGGTGGGATAATCCTTAgCTGTTCCTCCTC
 9G > C_24C > G sense: TAATACGACTCACTATA**GGGG**ATCGTCAGcTGGGATAATCCTTAgCTGTTCCTCCTC
 24C > T sense: TAATACGACTCACTATA**GGGG**ATCGTCAGGTGGGATAATCCTTAtCTGTTCCTCCTC
 9A_24T sense: TAATACGACTCACTATA**GGGG**ATCGTCAGaTGGGATAATCCTTAtCTGTTCCTCCTC

### Native gel electrophoresis

In vitro transcription templates for the mature *U8* snoRNA (136 nt), *U8* snoRNA variants (19G, 20T, and homodimer mutant) and a truncated *U8* snoRNA (nt 1–26 removed) were produced by PCR from the plasmid pRNA-hU8-GFP (Badrock et al. 2020) using the primers listed in the next section. PCR products were purified with the PureLink PCR Purification Kit (Invitrogen). In vitro transcription was performed using the T7 RiboMAX Express Large Scale RNA Production System (Promega) in a 20-uL reaction using 800 ng PCR product as template. PCR product was removed by RQ1 DNase treatment, and the reaction phenol extracted was then precipitated with ammonium acetate. *U8* snoRNAs were resuspended in water at a concentration of 0.4 µg/µL, then purity and size checked by denaturing polyacrylamide gel electrophoresis using UreaGel-6 (National Diagnostics). RNA was visualized using SYBR Green II RNA Gel Stain and a LI-COR Odyssey FC imager using the 600 channel. For native gel electrophoresis, *U8* snoRNAs were put in 50 mM Tris-Cl at pH 8, 100 mM NaCl, heated to 95°C, and cooled slowly to room temperature. *U8* snoRNAs were run at 4°C on a 6% native gel (37.5:1 Acrylamide:Bis-acrylamide) with TBM buffer (47.5 mM Tris-base, 47.5 mM Boric Acid, 5 mM MgCl_2_) at 120 V. RNA was visualized using SYBR Green II RNA Gel Stain and a LI-COR Odyssey FC imager using the 600 channel.

### PCR primers

 hU8_T7_F
 TAATACGACTCACTATAGGGGATCGTCAGGTGGGATAATCC
 mat_hU8_R
 AATCAGACAGGAGCAATCAGGGTGTTGCAAG
 phU8T7_19G_F
 TAATACGACTCACTATAGGGGATCGTCAGGTGGGATAATgCTTACCTGTTCCTCCTC
 phU8T7_20T_F
 TAATACGACTCACTATAGGGGATCGTCAGGTGGGATAATCtTTACCTGTTCCTCCTC
 phU8T7_delta-homo_F
 TAATACGACTCACTATAGGGGATCGTCAccTcccATAATggTTACCTGTTCCTCCTCCGG
 phU8T7_trunc_F
 TAATACGACTCACTATAGGGGTTCCTCCTCCGGAGGGCAG

### Software availability

The software used to perform these analyses can be downloaded from GitHub (https://github.com/gkudla/hyb).

The script used to calculate L (the overlap between arms of chimeras) is available at GitHub (https://github.com/gkudla/hyb/blob/master/bin/hyb_overlaps.awk) and as Supplemental Code.

## Supplementary Material

Supplemental Material
