# Supplementary material for: Global mapping of RNA homodimers in living cells
Source: Genome Res. 2022 May;32(5):956–67. doi: 10.1101/gr.275900.121 (PMC9104694; doi:10.1101/gr.275900.121)
Supplement: Supplemental Material [file supp_32_5_956__DC1.html]

Global mapping of RNA homodimers in living cells — Supplemental Material 

# Global mapping of RNA homodimers in living cells

## Supplemental Material

- Supplemental\_Figures.pdf
- Supplemental\_Code.zip
- Supplemental\_DatasetS1.xlsx
- Supplemental\_DatasetS2.xlsx
