# Supplementary material for: Global mapping of RNA homodimers in living cells
Source: Genome Res. 2022 May;32(5):956–67. doi: 10.1101/gr.275900.121 (PMC9104694; doi:10.1101/gr.275900.121)
Supplement: Supplemental Material [file supp_gr.275900.121_Supplemental_Figures.pdf]

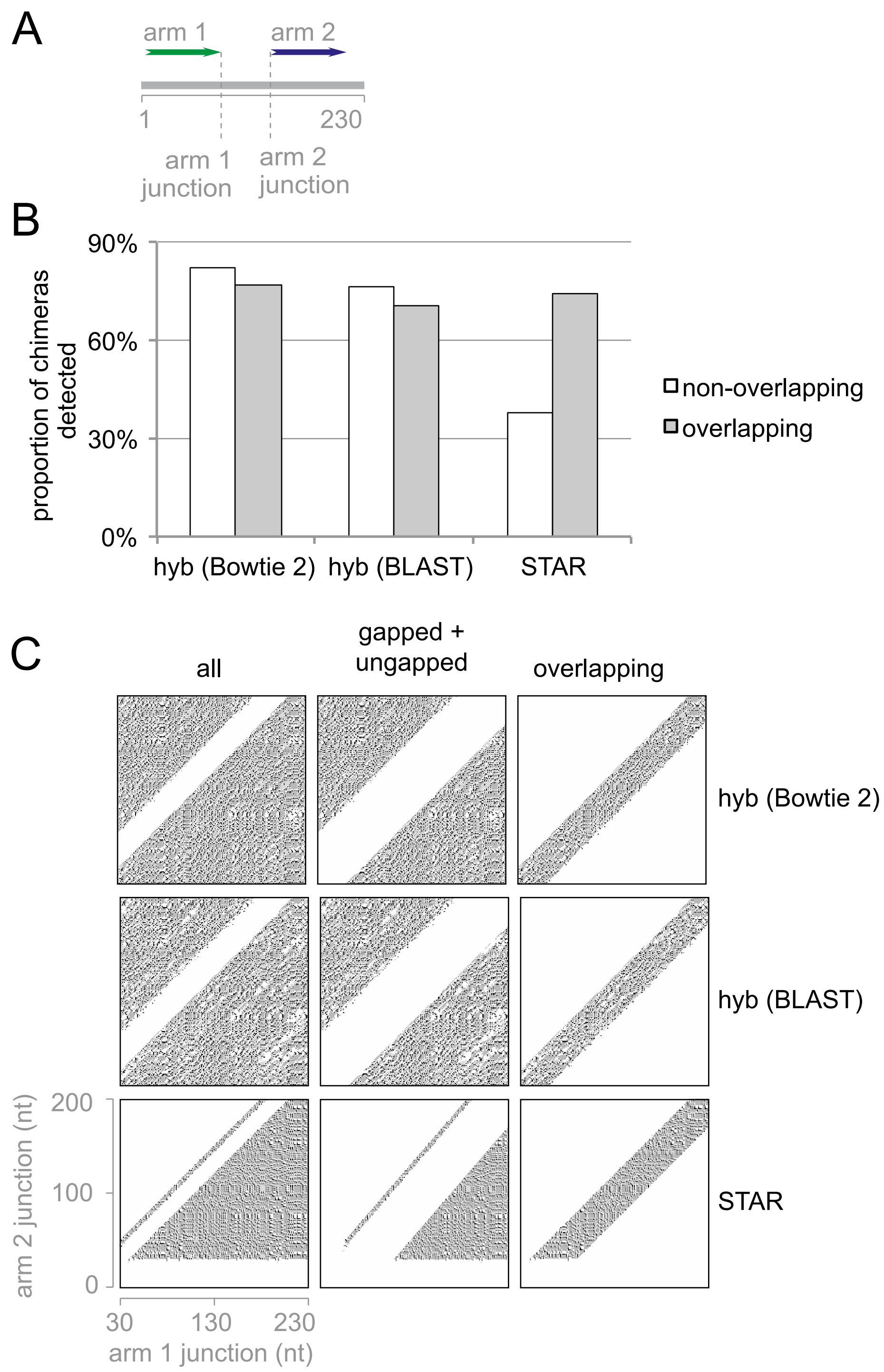

Figure S1. Benchmarking of chimera mapping tools on simulated chimeras.
(A) Design of benchmarking dataset: all pairs of 30-nt substrings of a 228-nt long yeast RNA sequence were concatenated, separated into overlapping and non-overlapping sets, and their junction positions were recorded.
(B) Proportion of simulated chimeras correctly identified as chimeric by hyb and STAR.
(C) Coverage heatmap of chimeric junction positions identified in the test dataset by hyb and STAR.

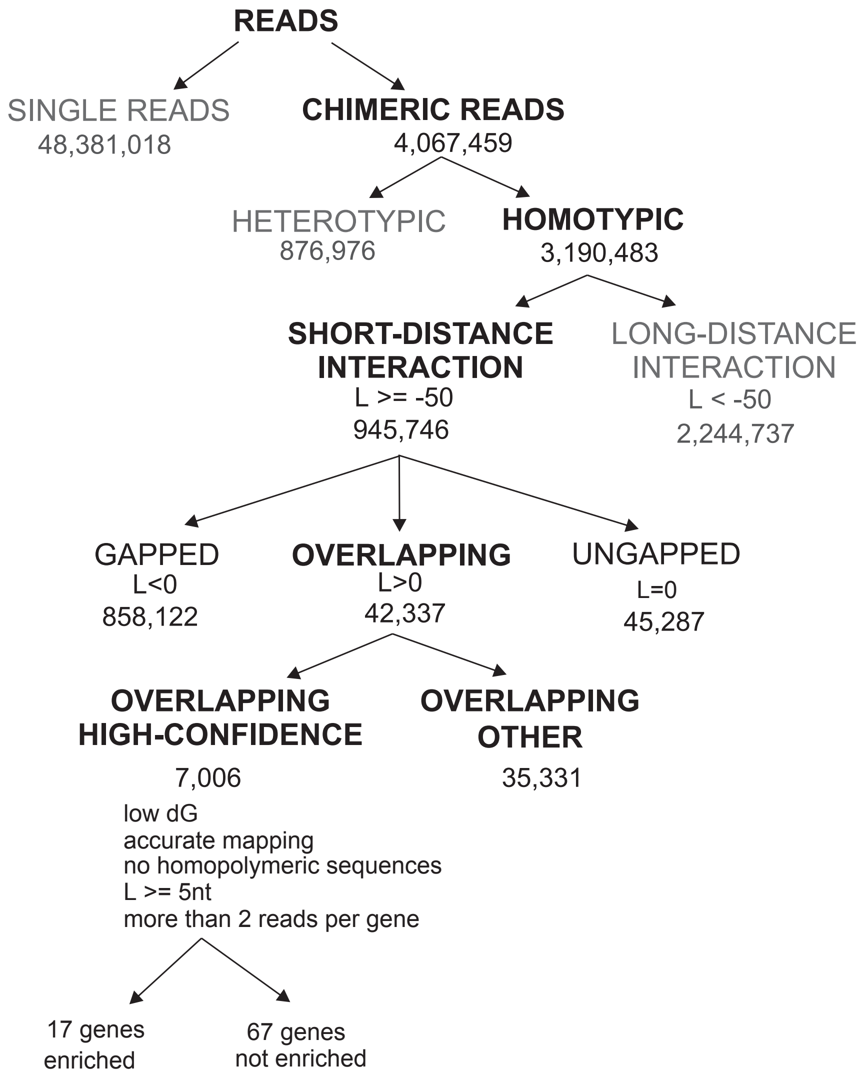


```
815092-1_5_ENSG_ENST_ZIKV-PE243-2015_virusRNA_10080_10124-815092-1_5_ENSG_ENST_ZIKV-PE243-2015_virusRNA_10033_10080
TGGATGACCACTGAAGACATGCTTGTGGTGTGGAACAGAGTGTGGGGGTTCCAACTGGGAGAACTACCTGGTCAATCCATGGAAAGGGAGAAT
TGGATGACCACTGAAGACATGCTTGTGGTGTGGAACAGAGTGTGG------------------------------------------------     10080   10124
---------------------------------------------GGGTTCCAACTGGGAGAACTACCTGGTCAATCCATGGAAAGGGAGAAT     10033   10080
((((((((((...........(((.((((.((((((...........)))))))))).))).......)))))).))))..............     (-21.9)
```

```
795982-1_5_ENSG_ENST_ZIKV-PE243-2015_virusRNA_3054_3079-795982-1_5_ENSG_ENST_ZIKV-PE243-2015_virusRNA_3022_3070
AAGGGAAAGGAGGCTGTACACAGTGAAGTGTGATCCAGCCGTTATTGGAACAGCTGTTAAGGGAAAGGAGGCTGT
AAGGGAAAGGAGGCTGTACACAGTGA-------------------------------------------------     3054   3079
--------------------------AGTGTGATCCAGCCGTTATTGGAACAGCTGTTAAGGGAAAGGAGGCTGT     3022   3070
...........((((((((((......)))))...))))).................................. .     (-12.8)
```

Figure S2. Filtering of chimeric reads to identify RNA homodimers.
(Top) Statistics of chimeric reads found in HEK293 PARIS (Lu, et al. 2016). (Bottom) Examples of overlapping chimeras from the Zika COMRADES dataset with positions of overlapping nucleotides indicated in red. The length of overlap is 1 nt in the top read and 17 nt in the bottom read. Therefore, only the bottom read will be considered to support a homodimer interaction.

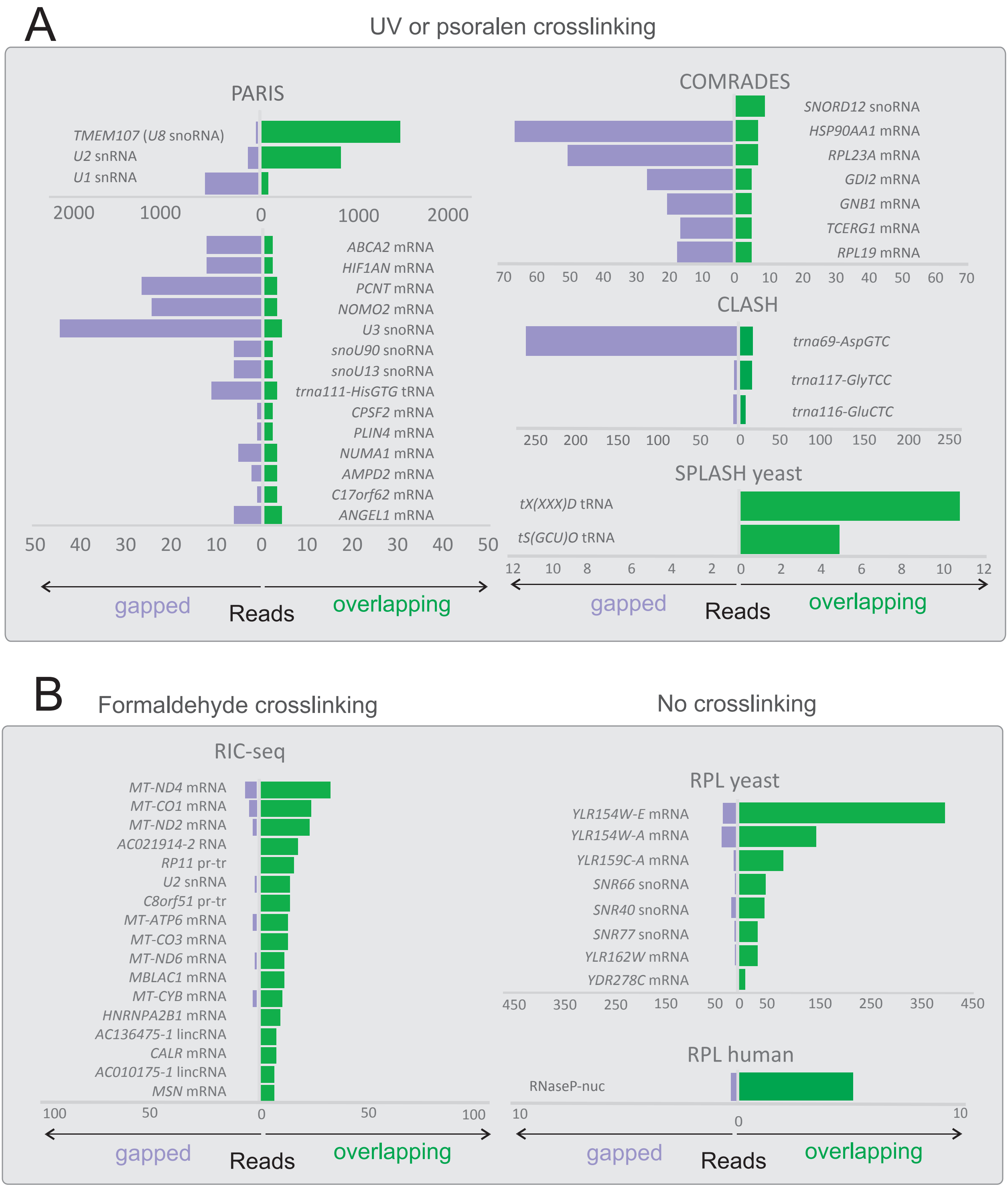

Figure S3. Genes with significantly enriched homodimers in various proximity ligation experiments.
(A) Counts of gapped and ungapped chimeras (purple) and overlapping chimeras (green) in individual genes in human PARIS data (Lu, et al. 2016); Zika COMRADES (Ziv, et al. 2018); human Ago1 CLASH (Helwak, et al. 2013); yeast SPLASH (Aw, et al. 2016)
(B) Counts of gapped and ungapped chimeras in human RIC-Seq (Cai, et al. 2020) and yeast and human RPL (Ramani, et al. 2015).

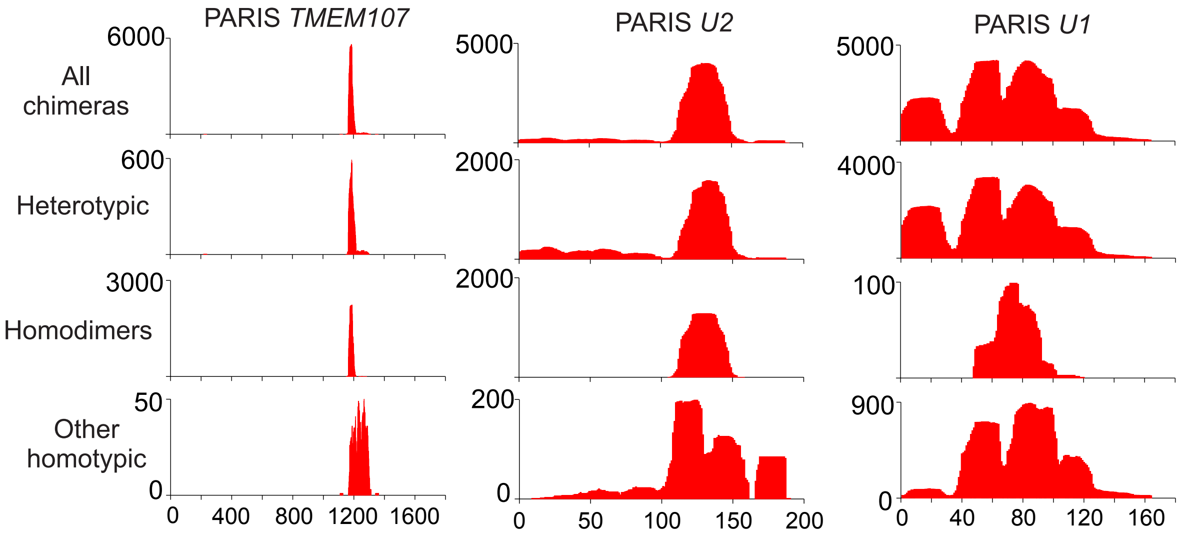

Figure S4. Coverage of chimeras in selected genes HEK293 PARIS data.
The X axis represents the position along the indicated gene, and the Y axis, chimera coverage. From top to bottom, the graphs represent, for each gene, the coverage of all chimeras, heterotypic chimeras, homotypic overlapping chimeras (homodimers), and other homotypic chimeras.

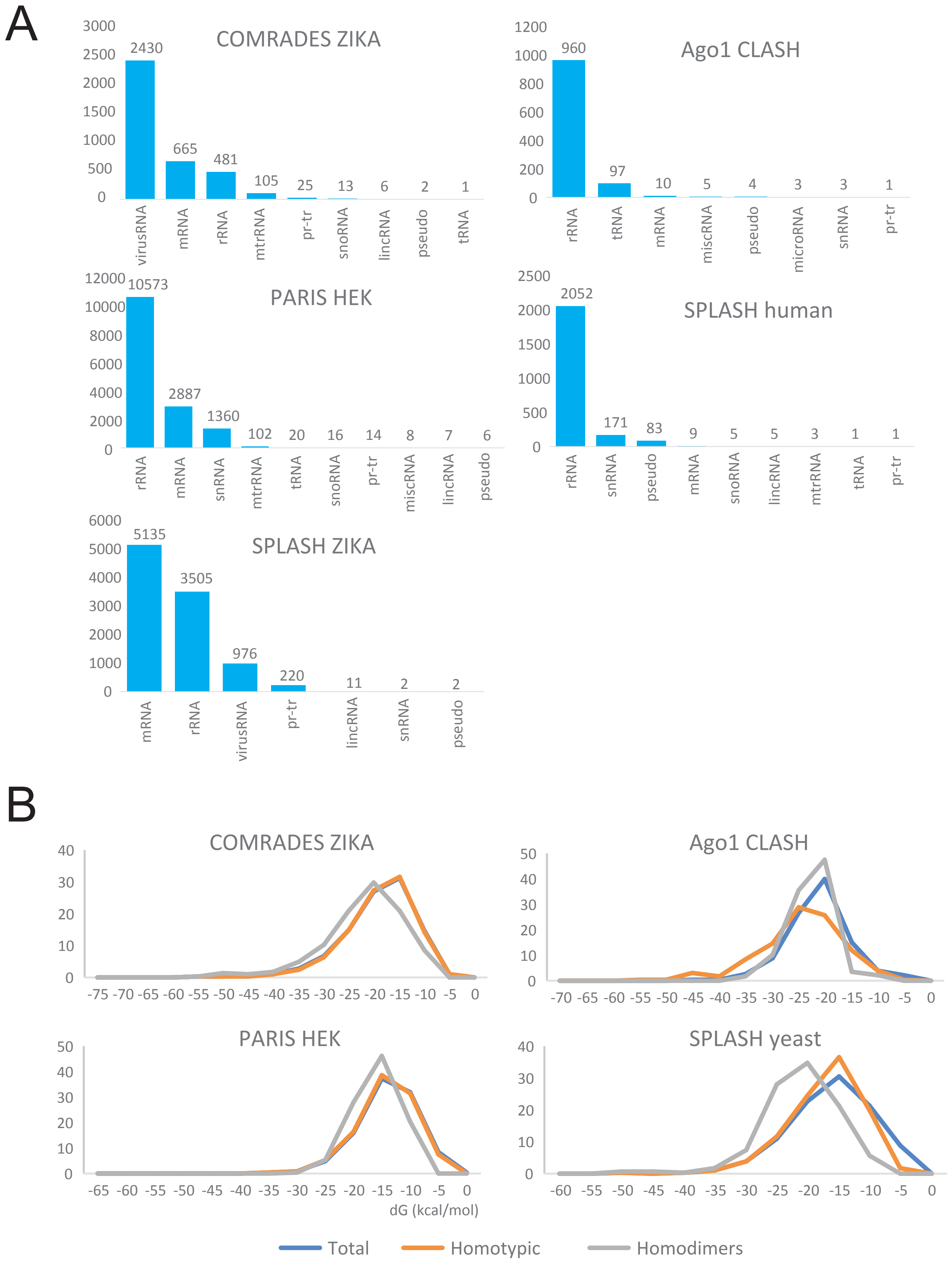

Figure S5. Statistics of homodimers in proximity ligation data
(A) Classification of genes with homodimers by RNA biotype.
(B) Distributions of predicted folding energies in all chimeras, homotypic chimeras, and overlapping chimeras indicative of RNA homodimers.

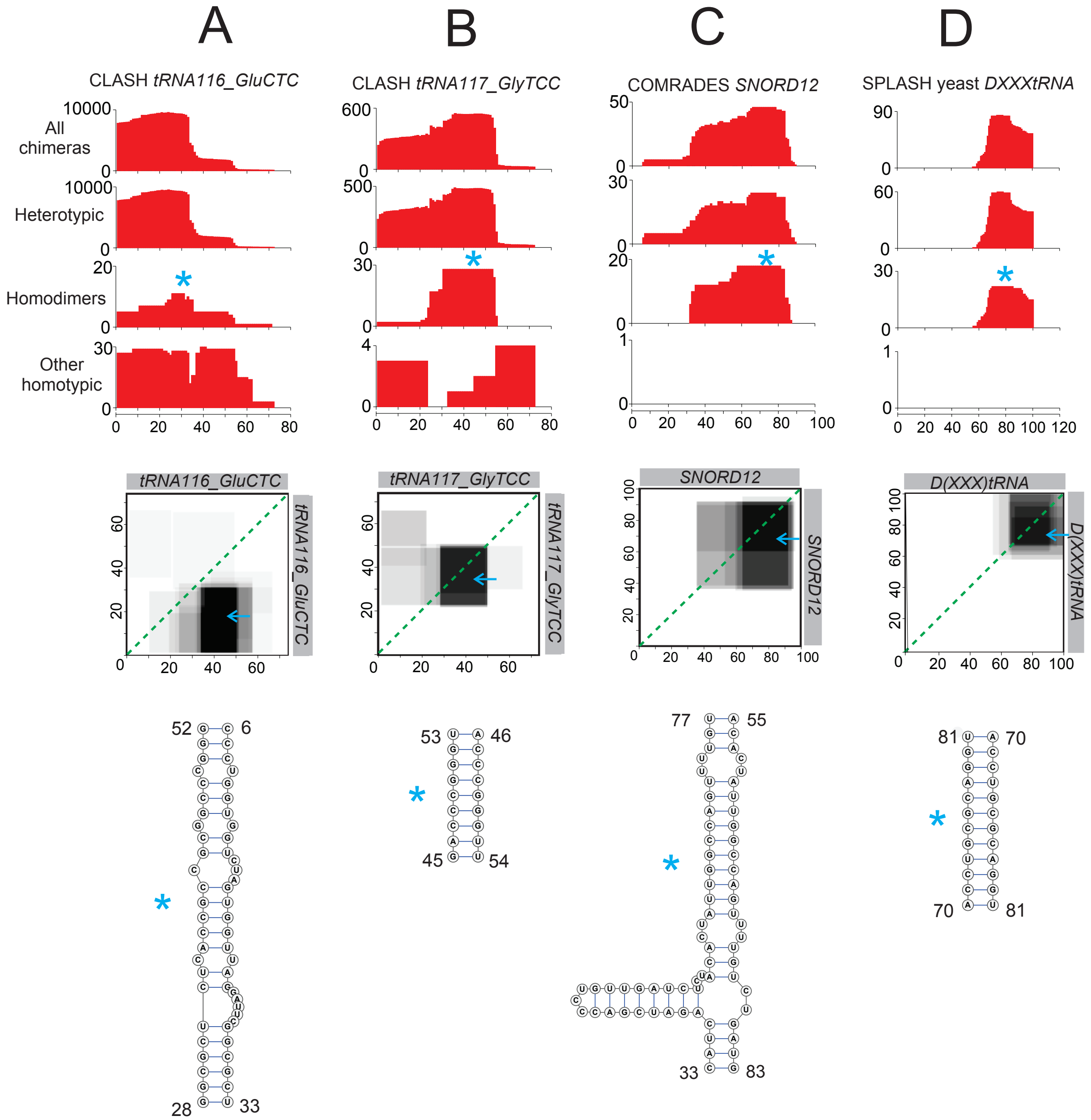

Figure S6. Example homodimers detected by UV- and psoralen-crosslinking methods
Data taken from CLASH (Helwak, et al. 2013), COMRADES (Ziv, et al. 2018), and SPLASH (Aw, et al. 2016).

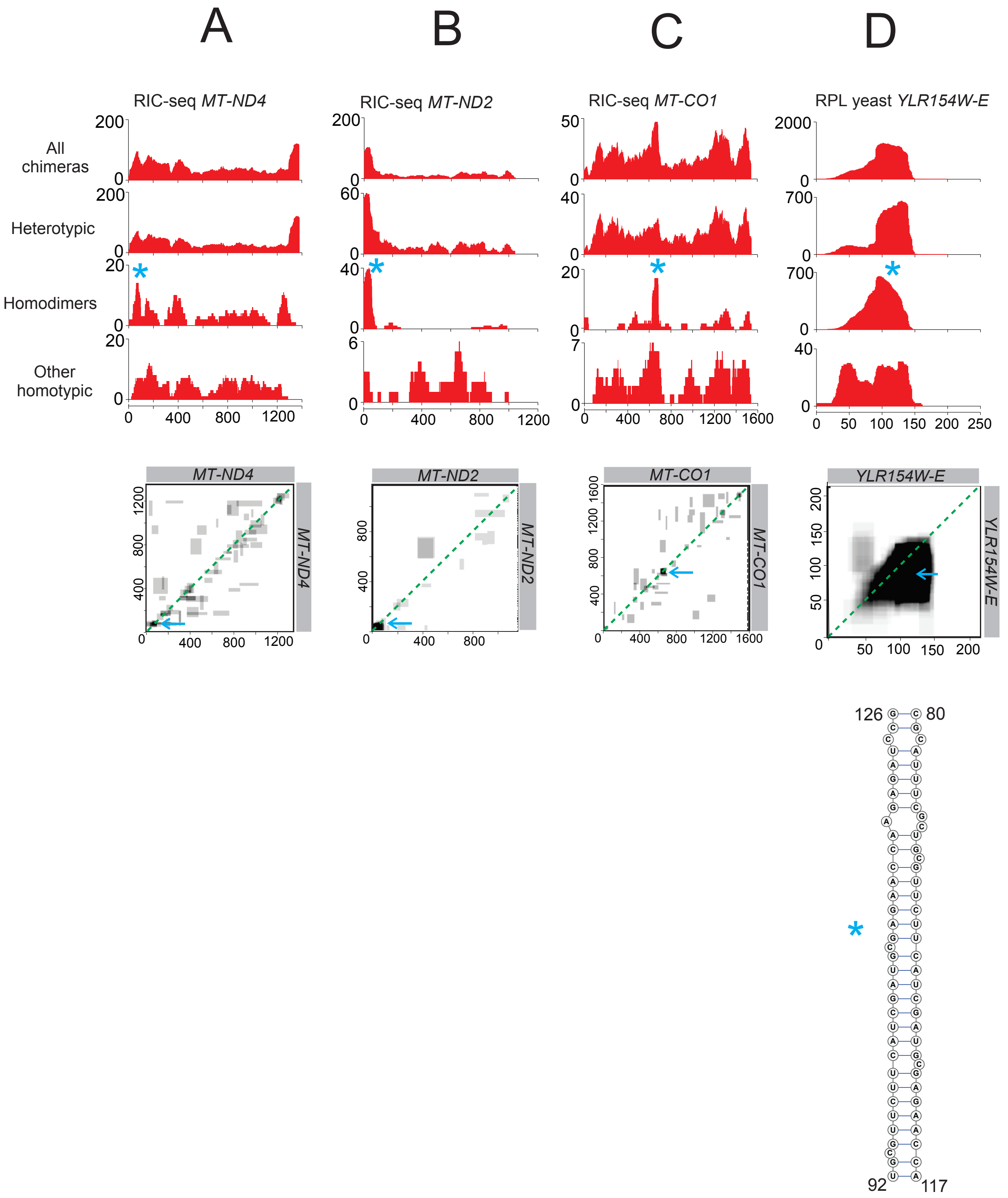

Figure S7. Example overlapping chimeras in formaldehyde and no-crosslinking experiments
Data are from RPL (Ramani, et al. 2015) and RIC-Seq (Cai, et al. 2020)

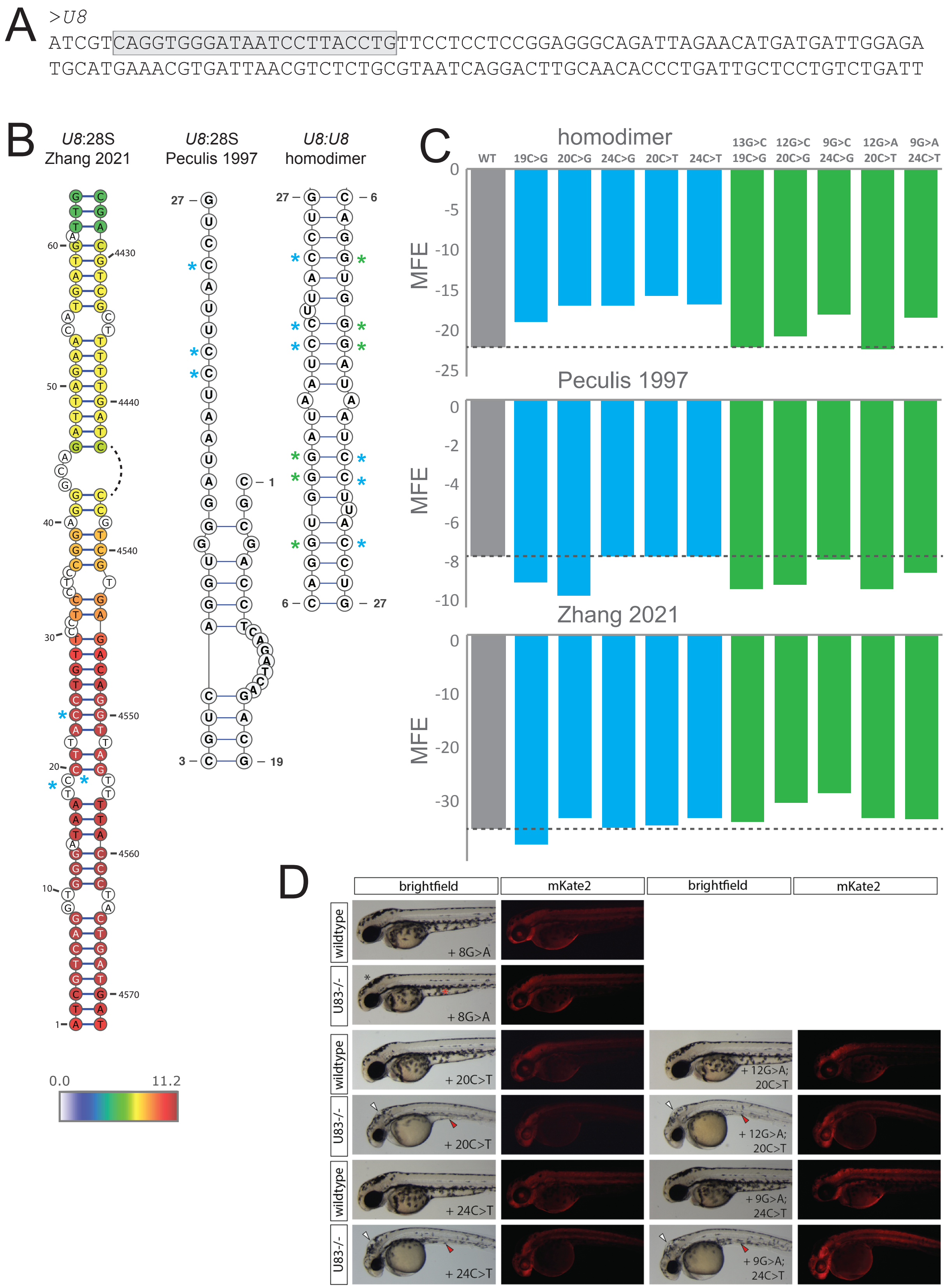

Figure S8. Effects of *U8* mutations on *U8*:*U8* and *U8*:28S interactions.
(A) *U8* sequence with homodimerisation domain indicated.
(B) *U8*:28S and *U8*:*U8* interactions described in the indicated studies. The colour bar represents the log2 coverage of chimeras supporting individual basepairs in the *U8*:28S interaction described in (Zhang, Li, et al. 2021).
(C) Effects of selected single and double mutations in *U8* on the predicted folding energy of *U8*:*U8* and *U8*:28S duplexes.
(D) Representative brightfield and fluorescent images of the indicated genotype and exogenous precursor *U8* snoRNA variants, as in Figure 5.

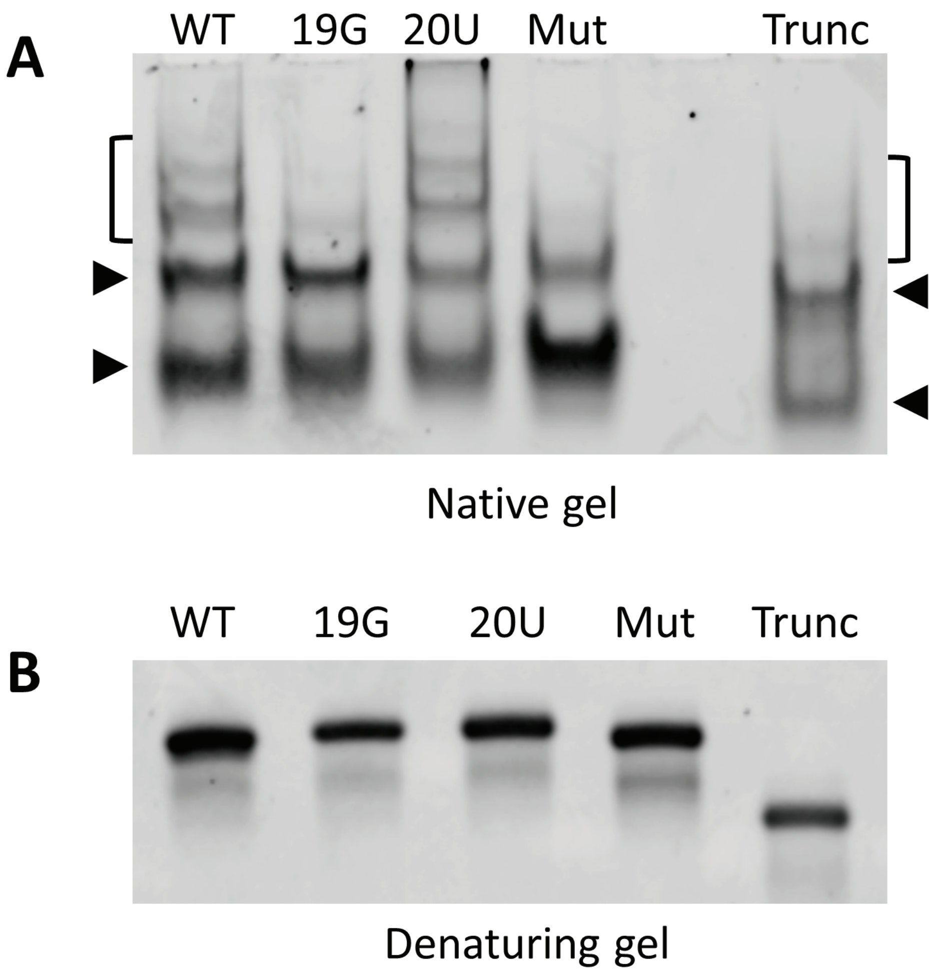

Figure S9. The *U8* snRNA forms high order complexes that are sensitive to mutation or removal of the predicted homo-dimer interaction region

A) Native gel electrophoresis reveals multiple conformers of the wild-type (WT) *U8* snoRNA. The larger conformers of the *U8* snoRNA, indicated by the brackets, are affected by homo-dimer mutations 19C>G (19G), 20C>U (20U), seven mutations in the homo-dimer region (Mut) and truncation of *U8* snoRNA by removal of the first 26 nucleotides (Trunc). Arrows indicate *U8* snoRNA conformers that do not change following mutation or removal of the homo-dimer domain.

B) Denaturing gel electrophoresis reveals that the *U8* snoRNAs used for native gel analysis are a single RNA species.
